# Developing Cryptic ACTL6B as Novel TDP‐43 Loss of Function Biomarker

**DOI:** 10.1002/alz.087164

**Published:** 2025-01-09

**Authors:** Margery JQ Chen, Katherine E Irwin, Koping Chang, Pei Jasin, Kerstin E Braunstein, Irika Sinha, Juan C Troncoso, Jonathan P Ling, Philip C Wong

**Affiliations:** ^1^ Johns Hopkins University, Baltimore, MD USA; ^2^ Johns Hopkins University School of Medicine, Baltimore, MD USA; ^3^ Johns Hopkins School of Medicine, Baltimore, MD USA

## Abstract

**Background:**

The nuclear clearance and cytoplasmic aggregation of splicing repressor TAR DNA/RNA‐binding protein‐43 (TDP‐43) occur in approximately 50% of Alzheimer’s disease (AD) cases and about 45% of frontotemporal dementia (FTD). However, it is not clear how early such mechanism occurs in AD and FTD as there is no method of detecting TDP‐43 dysregulation in living individuals. Since the loss of nuclear TDP‐43 leads to cryptic exon inclusion, we propose that cryptic exon‐encoded peptides may be detected in patient biofluids as biomarkers of TDP‐43 loss of function.

**Method:**

We developed antibodies against the TDP‐43‐dependent cryptic peptide within actin‐like protein 6B (ACTL6B) and characterized them through protein blot analysis using TDP‐43‐knockdown (KD) SH‐SY5Y neuroblastoma cell lysates. Next we performed immunofluorescent staining of patient brain tissues to determine whether cryptic ACTL6B can be found in patient neurons. We then developed a Meso Scale Discovery (MSD) ELISA using our cryptic ACTL6B antibody.

**Result:**

While the wild‐type (WT) ACTL6B antibody detected an expected band in both WT and KD SH‐SY5Y lysates, the cryptic ACTL6B antibody detected a band in only KD SH‐SY5Y. Using this antibody, we detected cryptic ACTL6B in FTLD and AD, but not control, brain tissues. The cryptic ACTL6B staining colocalizes with neurons that display TDP‐43 nuclear depletion and/or cytoplasmic aggregation. Using transfected SH‐SY5Y cells to overexpress either cryptic or WT ACTL6B, the MDS ELISA was sensitive and specific for cryptic ACTL6B, with 0.5 micrograms of lysate from SH‐SY5Y overexpressing cryptic ACTL6B showing MSD signal 5‐fold higher than 0.5 micrograms of lysate from SH‐SY5Y overexpressing WT ACTL6B (1546 vs. 290 units).

**Conclusion:**

The study provides evidence that our cryptic ACTL6B antibody is sensitive and specific for the ACTL6B cryptic peptide. This antibody could be used to determine TDP‐43 nuclear clearance/loss of function in patient brain tissues, providing a more sensitive approach to detecting TDP‐43 pathology than staining for TDP‐43 cytoplasmic aggregates. In the future, we will use our novel MSD ELISA to test the presence of cryptic ACTL6B in patients’ CSF and blood, which could aid in early‐stage disease detection.

